# Prevalence and Causes of Blindness and Visual Impairment in Sokoto State, Nigeria: Baseline Data for Vision 2020: The Right to Sight Eye Care Programme

**DOI:** 10.4103/0974-9233.80700

**Published:** 2011

**Authors:** Nasiru Muhammad, Rabiu M. Mansur, Adamu M. Dantani, Elizabeth Elhassan, Sunday Isiyaku

**Affiliations:** College of Heath Sciences, Usmanu Danfodiyo University, Sokoto, Nigeria; 1 National Eye Centre, Kaduna, Nigeria; 2 Ophthalmology Department, Usmanu Danfodiyo University Teaching Hospital, Sokoto, Nigeria; 3 Sightsavers, Kaduna, Nigeria

**Keywords:** Blindness, Eye Care Program, Nigeria, Vision 2020

## Abstract

**Purpose:**

To estimate the prevalence of low vision and blindness, identify the causes, and suggest policies for an effective eye care program based on 2005 data from Sokoto State, Nigeria.

**Materials and Methods:**

A stratified two-stage cluster sampling method was used to quantify the prevalence of blindness and the causes from 4 health zones in Sokoto State. Subjects were evaluated using a magnifying loupe, direct ophthalmoscope and torchlight. Data were collected based on the World Health Organization prevention of blindness coding for an eye examination. Prevalences with 95% confidence intervals (CI) were calculated and surgical coverage for causes of blindness was also analyzed.

**Results:**

The response rate was 91%. The prevalence of bilateral blindness was 1.9% (95% CI: 1.5–2.3%) ranging from 1.6% to 2.0% across the four health zones. The prevalence was 2.1% (95% CI: 1.6–2.6%) in males and 1.6% (95% CI: 1.1–2.1%) in females. The leading cause of bilateral blindness was cataract (51.6%), followed by uncorrected aphakia (20.9%) and glaucoma (11%). The prevalence of bilateral operable cataract was 1.9% (95% CI: 1.5–2.3%). The cataract surgical coverage (individuals with visual acuity <6/60) for the study was lower than the couching coverage (4.4% vs. 14.9%, respectively). Surgical coverage for trichiasis was 4.4%. The major barrier to cataract and glaucoma management was cost.

**Conclusions:**

The prevalence of blindness in Sokoto State is high yet the main causes are largely avoidable. Barriers can be reduced by appropriate health education regarding the eye care program and the provision of integrated, sustainable, affordable and equitable services.

## INTRODUCTION

VISION 2020 is a global initiative that is well underway worldwide.^1^ However, partners in this initiative need to increase their efforts particularly in underserved communities. There have been concerted efforts in advocacy and resource mobilization, joint planning, and strengthening national capacities through human resource development and technology transfer to developing countries.^2^ However, an obstacle to proper planning and successful implementation is the lack of reliable data on blindness in many underserved regions such as Sokoto State, Nigeria.

A population-based survey was undertaken in 2005 to generate data needed for planning an eye care program in Sokoto State (Sight Savers International, Kaduna, Nigeria. Sokoto state eye care program (SKTECP) 2005–2009 Project document. July 2005) [unpublished data]. Sokoto State, with a population of 3.5 million, is located in north-western Nigeria and lies within the Sahel Savannah ecoregion that is interspersed with some desert areas. For effective planning and service delivery, the Local Government Areas (LGAs) in the State are grouped into four health zones: Yabo, Wurno, Dange-Shuni, and Gwadabawa. This paper reports baseline data used to facilitate the planning of the Sokoto State Eye Care Programme. Prior to this study, no national or state-wide survey had been undertaken. The estimated national prevalence (regardless of age) of blindness for Nigeria was projected to be 1.3% (Federal Ministry of Health, Nigeria. Strategic plan VISION 2020 [unpublished]: The Right to Sight Plan for Nigeria 2007–2011. Nov. 2006:17).

The objectives of the study were to (i) estimate the prevalence and magnitude of blindness and low vision in Sokoto State; (ii) determine the causes of blindness and low vision in the state; (iii) estimate the prevalence of refractive error in the State; (iv) estimate the magnitude of operable cataract in the state; and (v) estimate the ophthalmic surgical coverage and determine the barriers to uptake the ophthalmic services for some causes of blindness in Sokoto State.

## MATERIALS AND METHODS

Ethics approval was obtained from the ethics committee of the National Eye Centre, Kaduna, Nigeria. An approval for the conduct of the survey was obtained from the Sokoto State Ministry of Health and the respective local health authorities and community leaders. Verbal consent was obtained from the participants or their guardians. This study was performed in accordance with the tenets of the declaration of Helsinki.

A population-based cross-sectional survey of individuals of all ages was conducted from November 2005 to December 2005. A modified World Health Organization (WHO) prevention of blindness (PBL) eye examination record was used for data collection.^3^

A minimum sample size of 5303 persons of all ages was determined using the Epi info version 6.0 software (US Centre for Disease Control/WHO) based on the following parameters:

- Total populations of Sokoto State 3,571,843 (2005 Projection) (National Population Commission, Abuja, Nigeria. Nigeria census 1991. Projected to 2005) [unpublished]
- Assumed prevalence of blindness 1.5% (based on blindness prevalence of 2% in one of the LGAs in the state^4^)
- Worst acceptable result ±0.4%, design effect 1.5 (a cluster randomized study), confidence interval (CI) 95% (*P* <0.05) an additional 10% to cover the nonresponse rate.

### Data collection and statistical analysis

A stratified multistage cluster sampling technique was used for the survey. Stratification was based on the health zones. The number of individuals selected for inclusion in each zone was based on the percentage of the population living in the zone. The proportion was a representative sample of individuals for each zone.

In each zone, the study population was drawn by a two-stage cluster random sampling technique with probability proportional to size (PPS). In the first sampling stage, 72 communities (clusters) were randomly selected across the four health zones, of which 19 clusters (28%) were selected from the Wurno zone; 18 clusters each (24.5%) from the Gwadabawa and Dange-Shuni zones; and 17 clusters (23%) from Yabo zone. In the second sampling stage, 80 individuals of all ages were randomly chosen in each of the selected clusters. The selection of clusters in rural and urban settlements was based on a previous study in a nearby community.^5^ Individuals that stayed for at least 6 months in the community were included in the survey.

Two teams each comprising of one ophthalmologist, two ophthalmic nurses, and one community health extension worker (CHEW) conducted the surveys. Two-day trainings and a pilot study were conducted in a nearby nonselected community to familiarize the teams with the instruments and procedures for the survey. The training emphasized visual acuity (VA) assessment and the operational definitions for the causes of visual impairment in order to reduce intra/interobserver variations. Each team member had his/her role specifically defined and demonstrated and was required to practice it prior to study initiation. The ophthalmologists compared records in cases of disagreement to ensure the appropriate use of the standardized operational definitions. Although a Kappa analysis was not performed, the results were in agreement in more than 90% of the examined subjects; those with a different cause of blindness/low vision by the ophthalmologists were discussed and the cause of the disparity was identified to ensure use of standardized definitions. The enumeration process is described in our previous paper.^5^ Chi-square analyses were conducted and a *P* <0.05 was considered statistically significant and 95% CI were calculated.

### Vision testing

Ophthalmic nurses recorded demographic data of each participant and then tested the presenting VA monocularly, using the Snellen E chart placed at a distance of 6 m. The details of the VA assessment in adults and schoolchildren have been previously described.^5^ All individuals were then referred for basic eye examination by the ophthalmologist.

Children less than 5 years of age and subjects in whom VA could not be determined were classified as either “believed blind” or “believed not blind” in each eye by the ophthalmologists. Aphakes were identified by the ophthalmologist and VA retested with aphakic glasses (+10 D lens).

The ophthalmologist used a penlight, 2X magnifying loupe, and a direct ophthalmoscope for examination of the posterior segment. The detailed ophthalmic examination has been previously described.^5^ The operational definitions for the disorders were based on WHO PBL coding instructions for the eye examination record form except for the operational definition of Glaucoma, which was defined as a cup:disc ratio of 0.8 and more.^6^ Blindness was defined as presenting vision of less than 3/60 in the better eye and low vision as presenting vision of less than 6/18 to 3/60 in the better eye.^7^ Uncorrected refractive error was defined as VA improving to 6/18 or better with pinhole. Ranking of disorders causing visual impairment/blindness was conducted as recommended by WHO PBL.^6^ Enquiries were subsequently made as to why individuals with trachomatous trichiasis, operable cataract (VA <6/60), and glaucoma had not undergone treatment/surgery. The response(s) from the participants were classified as barrier(s); up to four reasons are marked per subject.

All data were recorded into a modified WHO blindness survey form. Persons identified with treatable blindness or low vision were referred for further assessment and treatment. Survey participants were offered chloramphenicol eye drops or tetracycline eye ointment if appropriate.

Ophthalmologists rechecked the data periodically to complete missing information. Periodic discussions were held by the ophthalmologists to ensure consistent use of uniform operational definitions and examination procedures. Repeat visits were conducted to examine absentees.

Data entry and analysis was performed by a statistician using a pre-designed program in Epi-info version 6.0 software.

## RESULTS

Ninety-one percent (4848 persons) of the sample size were examined and 2.4% (129 persons) refused examination. Females constituted the majority (67%) of individuals who refused examination. The response rate ranged from 85% to 93% among the different health zones. Reasons for refusal among women were mainly absence of or refusal by the spouse. Table 1 shows the age distribution of the examined population. Persons 0–9 years constituted the highest percentage of the study participants (37.5% of 4848). Females constituted 43.1% of the study sample.

**Table 1:** Age distribution, prevalence of bilateral blindness, and low vision in a study sample from Sokoto State, Nigeria

Age group	Examined	No. Blind (n)	%	No. low vision (n)	%
0-9	1816	1	0.1	0	0
10-19	923	5	0.5	11	1.2
20-29	589	5	0.8	33	1.2
30-39	473	5	0.2	42	8.9
40-49	396	3	0.8	77	19.4
50-59	293	14	4.8	91	31.1
60-69	208	30	14.4	105	50.5
70-79	106	20	18.9	66	62.3
≥80	44	12	27.3	17	38.6
Total	4848	91	1.9	442	9.1

No.: number, %: prevalence

The prevalence of bilateral blindness (91 individuals) was 1.9% (95% CI: 1.5–2.3%) [Table 1]. The prevalence of blindness across the zones ranged from 1.6% to 2.0%. The prevalence among males was 2.1% (95% CI: 1.6–2.6%) and 1.6% (95% CI: 1.1–2.1%) in females. The difference in the prevalence of blindness between genders was not statistically significant (*P*>0.79). The higher refusals among females may also have influenced the rates. The gender-adjusted prevalence of blindness for the target population and the estimated number of blind individuals in the population are presented in Table 2.Although age- and gender-adjusted rates and estimations are desirable, they could not be calculated due to inadequate separation of target population data by age groups.

**Table 2:** Prevalence and magnitude of bilateral blindness and low vision among male and females subjects from Sokoto State, Nigeria

	Population examined (n)	Persons in sample (n)	Blind in sample n (%)	Low-vision n (%)	Blind in population n	Low-vision in population
Male	1,775,666	2758	58 (2.1)	251 (9.1)	37,228	161,585
Female	1,796,177	2090	33 (1.6)	191 (9.1)	28,738	163,452
Total	3,571,843	4848	91 (1.9)	442 (9.1)	65,966	325,037

%: prevalence

The prevalence of low vision was 9.1% (95% CI: 8.3–9.9%). A total of 171 individuals were blind in one eye. Thus the prevalence of unilateral blindness was 3.5% (95% CI: 3–4%) and was significantly higher among males (4.1%) compared to females (2.7%) (*P* = 0.007).

**Figure 1:**
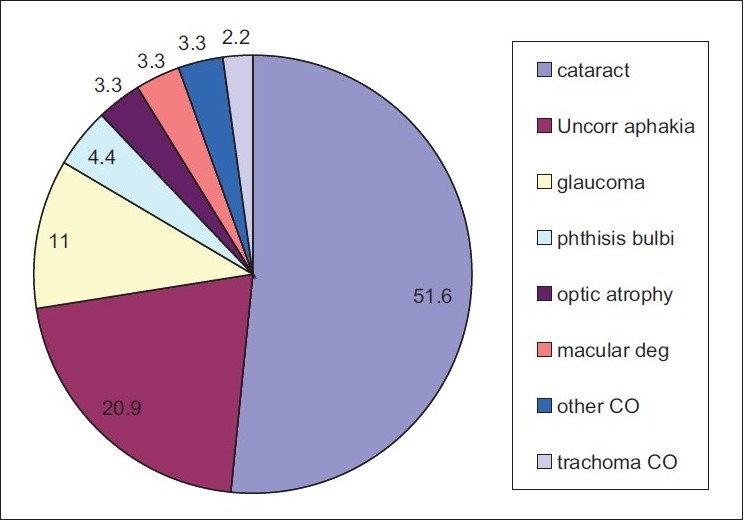
Causes of bilateral blindness (%)

**Figure 2:**
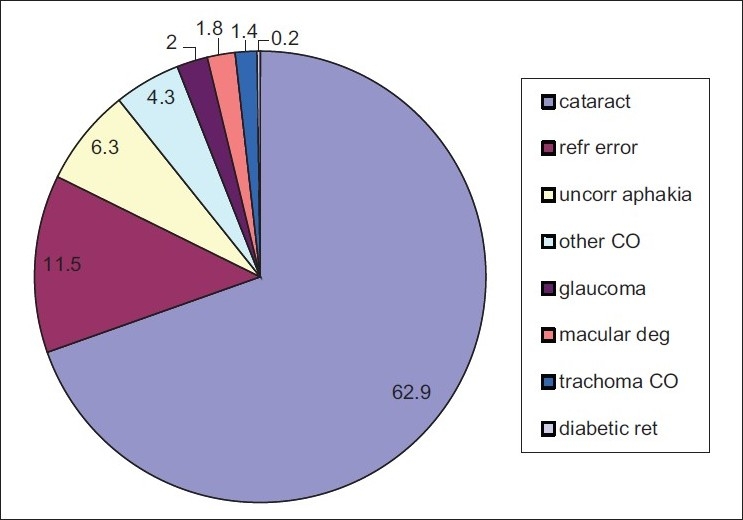
Causes of low vision (%)

Among those with bilateral blindness and low vision, we analyzed the proportion of principal causes [Figures 1 and 2, respectively].

The leading causes of unilateral blindness were cataract (53.8%), nontrachomatous corneal opacity (12.9%), and uncorrected aphakia (10.5%). Nontrachomatous corneal opacities were mainly due to trauma, infectious keratitis, and measles/xerophthalmia.

The prevalence of uncorrected refractive error was 1.5% (95% CI: 1.2–1.8%). The prevalence seems to increase with age-group up to the eighth decade of life with a significant difference between the age groups (*χ^2^*=160.15, df 8, *P*=0.0000). Extrapolating the prevalence of uncorrected refractive error to the population showed that between 42,108 and 63,162 individuals required spectacles to maximize their visual potential.

Ninety-three individuals had bilateral operable cataract (VA<6/60) and an additional 146 had unilateral operable cataract. Thus the prevalence of operable bilateral cataract was 1.9% (95% CI: 1.5–2.3%) and unilateral cataract was 3% (95% CI: 2.5–3.5%). Extrapolating this prevalence of bilateral operable cataract to the target population suggests that 52,635 to 80,707 individuals require bilateral cataract surgery and an additional 87,725 to 122,815 individuals require unilateral cataract surgery.

Among the participants, 13 individuals had undergone bilateral cataract surgery and 44 individuals had undergone bilateral couching. The cataract surgical coverage (persons) (CSC) (for vision of <6/60) was 4.4% (4.9% males; 3.6% females) while the coverage for couching was 14.9%.

Two individuals had undergone previous lid surgery for trachomatous trichiasis (TT) and 43 others had TT. The trichiasis surgical coverage (TSC) (persons) was 4.4%.

**Figure 3:**
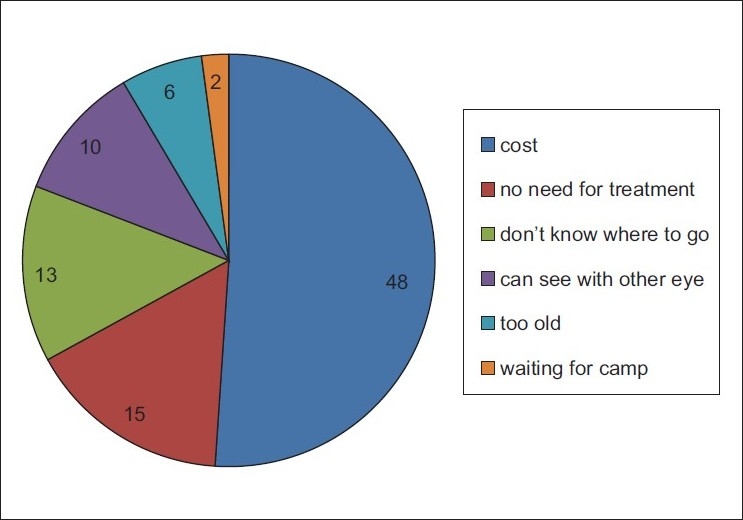
Barriers to cataract surgery (%)

The outcome of the study to understand the barriers to uptake of cataract surgeries are presented in Figure 3. The majority (48.4%) of individuals with operable cataract reported that they could not afford the cost of surgery.

Couching as treatment for cataract was preferred by 3.4% of those with cataract. Among those who had undergone couching, 22% stated that the prohibitive cost of cataract surgery was the reason to opt for couching and another 22% stated that they did not know where to undergo cataract surgery.

Thirty-six percent of those with TT did not know where to go for treatment, 19.4% did not feel the need for surgery, 16.7% reported that they could not afford the cost of surgery, and 13.8% were not aware of their problem. “Can still see with eye” was given as a reason for not seeking medical attention by another 5.6% of individuals with TT.

Nine individuals (31%) with unilateral or bilateral glaucoma said that they “cannot afford cost of the treatment,” eight individuals (27.6%) were not aware they have glaucoma, and seven individuals (24.1%) did not know where to go for treatment. Another two individuals (6.9%) reported that they knew where to go for treatment; however, the service was too far.

## DISCUSSION

The response rate in our survey is 93%; however, there may be an over-representation of males in this study due to the relative lack of participation from females. Refusal by females was mainly due to their husbands not consenting to the examination. This is a potential problem as there may be an inordinate number of males who receive ophthalmic care in this male-dominated Muslim population.

The prevalence (1.9%) of blindness (bilateral) for the population was higher than that estimated for the country (1.3%) (Federal Ministry of Health, Nigeria. Strategic plan VISION 2020: The Right to Sight Plan for Nigeria 2007–2011. Nov. 2006:17). The combined age and gender-adjusted prevalence rates were not analyzed as the census data available do not contain the age groups of the population.

The prevalence is also higher than that reported in a nearby state of Kaduna (0.6%).^8^ The presence of an established eye care program for over a decade with one of the largest concentration of eye health resources in terms of personnel and facilities in Nigeria explains this difference in Kaduna state. In Sokoto state, there is rudimentary and at best, intermittent eye care services with one ophthalmologist (also an administrator) in a referral hospital. The major barriers in this community were “cost” and a lack of awareness regarding ophthalmic care and services.

The prevalence of blindness was higher in our study compared to that reported in Malaysia (0.28),^9^ Gambia (0.7%),^10^ Ethiopia (0.85),^11^ South Africa (1.0),^12^ and in a population of southern Nigeria (1.18%).^13^ However, it was lower than that reported in a Sudanese population (4.1%).^14^ The Yabo and Wurno health zones had the highest rates of blindness (2.0%); it is therefore appropriate that programs prioritize these two zones for implementation of an action plan for eye care.

Our outcomes indicate that the major causes of blindness in Sokoto State are avoidable in over 80% of the bilaterally blind and 90% of the unilaterally blind individuals. Similar findings have been reported in Gambia (92%), Ethiopia, and Sudan.^10^,^11^,^14^ The avoidable causes in our study are, however, higher than the avoidable causes reported in the onchocerciasis endemic population of Kaduna state (51.3%), South Africa, and Malaysia where nonavoidable causes were the significant cause of blindness.^8^,^9^,^12^ Treatable causes such as cataract and uncorrected aphakia were responsible for 72.5% and 64.2% of bilateral and unilateral blindness, respectively.

The causes of low vision in Sokoto State were also largely avoidable. Curable causes (cataract, refractive error, and uncorrected aphakia) were responsible for 80.7% of low vision. A similar trend was reported for a Malaysian population.^9^ In contrast, trachoma and cataract were the major causes of low vision in Sudan.^14^

With a CSC (person) lower than the couching coverage (4.4% vs. 14.9%), the program faces the dual challenges of increasing awareness among the population and providing accessible and affordable eye care services. The CSC was similar to the findings in nearby Katsina State where a CSC (persons) of 4% was reported (in persons 40 years and older) and a couching coverage of 18%; the two neighboring states share similarities in terms lack of eye care resources and services.^15^

The simple provision of aphakic glasses is necessary as uncorrected aphakia was the second leading cause of blindness (20.9%) and among an estimated 14,036 persons (0.4%) required spectacles. This is a cost-effective remedy to treat preventable blindness and it can be freely available to aphakes.

Despite the limitations in the operational definition of uncorrected refractive error, the trend in refractive error was significantly higher in the more urban health zones (*P* = 0.001). In rural health zones, provision and use of spectacles is urgently needed. A population of presbyopes among the study population was willing to pay for reading glasses.

As only an advanced glaucoma causing visual disability was detected in our study, the magnitude of glaucoma could be higher due to the surreptitious nature of this disease and lack of screening for early cases in our study. A comprehensive eye examination incorporating standard glaucoma screening and a programmed approach to address glaucoma is needed.

Trachomatous corneal opacity was responsible for 2.2% of both bilateral and unilateral blindness. The trichiasis surgical coverage (TSC) was also low, indicating that the current community-based eyelid surgical services need to be intensified to close the gap between the demand and the current service. Even TSC in this study is higher than that reported in districts of Southern Sudan (0.5–6%), it needs considerable improvement.^16^ Major barriers for the uptake of lid surgery are patient-related and hence health education regarding treatment of TT and TCO is recommended.

The burden of blindness and visual impairment in Sokoto State was higher than the national average of 1.3% and the causes were largely avoidable. The eye care program should intensify public education of eye diseases and the need for eye care. It should also focus on providing accessible, accountable, affordable, and sustainable comprehensive eye care services.
